# Neck dissection in pT1 maxillary squamous cell carcinoma: Necessity or overtreatment?

**DOI:** 10.1007/s10006-025-01447-y

**Published:** 2025-10-11

**Authors:** Max Lukas Linderkamp, Pia Maria von Moltke, Stephan Alexander Bettag, Anij von der Gracht, Fritjof Lentge, Philipp Jehn, Michael-Tobias Neuhaus, Nils-Claudius Gellrich, Philippe Korn

**Affiliations:** Department for Oral and Maxillofacial Surgery, Hannover Medical School, Carl- Neuberg-Straße 1, Hannover, 30625 Germany

**Keywords:** Oral squamous cell carcinoma (OSCC), Neck dissection (ND), Cervical lymph node metastases (cLNM), Maxillary squamous cell carcinoma (MSCC)

## Abstract

**Purpose:**

Squamous cell carcinoma (SCC) is the most common malignancy of the oral cavity. Despite cN0 staging, elective neck dissection (END) is often performed to detect occult cervical lymph node metastases (cLNM). Maxillary SCC (MSCC) generally exhibits similar metastases rates to intraoral SCC in other locations. However, due to the rarity of T1 MSCC, the current guidelines of the German Society for Oral and Maxillofacial Surgery do not provide a clear recommendation for neck dissection (ND) in these cases, as existing data are limited.

**Methods:**

A retrospective analysis was conducted on all cases with SCC of the superior level of the oral cavity diagnosed at Hannover Medical School over the past 20 years (*n* = 225). cLNM in pT1 cases (*n* = 36) were specifically examined.

**Results:**

In pT1 MSCC cases confined to the alveolar process and hard palate, cLNM was detected in only one patient (1/25, 4.00%). In contrast, a significantly higher occurrence of cLNM was observed in pT1 SCC of the soft palate (4/11, 36.36%, *p* [Fisher] = 0.023).

**Conclusion:**

In accordance with current guidelines, ND may not be necessary for pT1 MSCC. However, if SCC infiltrates the soft palate, ND should be considered. Given the rarity of pT1 MSCC, further studies with larger datasets are needed to establish definitive recommendations.

## Introduction

Worldwide, approximately 2% of all cancer occur in the oral cavity and lips; in 2020, nearly 380,000 people were diagnosed with oral cancer, and approximately 180,000 died from the disease [1]. Squamous cell carcinoma (SCC) is the most common type, accounting for around 90% of cases [2–6]. The five-year overall survival rate for oral SCC is approximately 54–65% [7–9].

Maxillary SCC (MSCC) is relatively rare, representing only 3.5–11% of all oral SCC cases [10–13]. However, it is associated with a poorer prognosis compared to SCC in other oral cavity locations [7, 9].

Oral SCC frequently exhibits cervical lymph node metastases (cLNM), which primarily occur ipsilaterally but can also present contralaterally or bilaterally [14, 15]. MSCC metastasizes to cervical lymph nodes in approximately 35% of cases, a rate comparable to SCC in other oral sites [16–18]. To guide decisions regarding the necessity and extent of neck dissection (ND), thorough staging is conducted before tumor resection. This includes palpation of cervical lymph nodes, and radiological imaging - typically sonography and/or contrast-enhanced spiral computed tomography versus magnetic resonance imaging. Additionally, further staging investigations are performed to assess distant metastases.

Elective neck dissection (END) is performed to detect possible occult cLNM in patients staged as cN0 [19]. Studies have demonstrated that END improves overall and disease-free survival in oral SCC cases (all sites, T1/T2, without midline crossing) after a three-year follow-up [20]. A survival benefit has also been observed for END in MSCC, with occult cLNM detected in up to 26% of MSCC cases [18, 21].

While END is widely recommended for other oral SCC subsites, guidelines for MSCC remain unclear [20, 22]. ND ensures the removal and detection of occult cLNM, with the pathological nodal (pN) status serving as a crucial prognostic factor. The pN status is considered the most reliable indicator of patient outcomes and plays a key role in decisions regarding adjuvant treatment [8]. However, ND can be associated with significant morbidity, including nerve damage, restricted mouth opening and swallowing disorders [23].

The necessity of END for T1 MSCC remains controversial [24]. According to the current S3 guideline, Diagnosis and Treatment of Oral Cavity Carcinoma, published by the German Society for Oral and Maxillofacial Surgery (DGMKG), no definitive recommendation can be made against level I–III ND and in favor of a “wait-and-see” approach in MSCC. However, expert consensus suggests that in cases of cT1 cN0 MSCC confined to the alveolar process and hard palate with an invasion depth of < 3 mm, level I–III ND may be omitted if close follow-up is ensured [15, 25].

Further complicating matters, the implementation of ND in Germany remains inconsistent, with no standardized approach regarding staging, surgical extent, or unilateral versus bilateral dissection [26]. Due to the limited data available on END in T1 MSCC, the aim of this paper was to generate robust evidence to support evidence-based decision-making.

## Materials and methods

All pT1-classified SCC of the superior level of the oral cavity diagnosed at Hannover Medical School (MHH) within a period of 20 years (between January 1 st, 2003, and January 1 st, 2023), were analyzed. They were subsequently separated into OSCC of the hard palate, alveolar process and maxillary gingiva (≙ MSCC) and OSCC of the soft palate. The relevant data were retrieved from the hospital’s data warehouse. Collected variables included patient gender, date of diagnosis, tumor localization, results of cervical staging examination, treatment details - particularly regarding cervical lymphadenectomy - and the histopathological TNM classification of both the primary tumor and resected cervical lymph nodes. The TNM classification followed the guidelines of the Union for International Cancer Control (UICC). pT classification for T1 carcinomas did not change significantly during the examination period. In contrast to the 6th and 7th TNM classification the 8th edition, that the current guideline (2021) is based on, includes depth of invasion ≤ 5 mm for T1 carcinomas.

SCC occurring at the transition between the hard and soft palate were classified as soft palate SCC, in accordance with guideline recommendations, which are limited to tumors of the alveolar process and hard palate. The study examined the number of patients with pT1 SCC of the superior level of the oral cavity who presented with cLNM. Additionally, follow-up duration was assessed to determine whether cLNM developed over time. For patients who continued tumor follow-up beyond January 1 st, 2023, this date was recorded as their last follow-up appointment. However, not all patients had complete follow-up data, as some did not attend regular check-ups, while others opted for local follow-up care, closer to their main place of residence.

A prerequisite for study inclusion was prior patient consent for the processing of personal data, which was obtained in all cases. Two patients developed a secondary carcinoma more than one year after the initial diagnosis. One in the contralateral maxilla and another at a different maxillary site. As both were second primary carcinomas of the maxilla, they were included as separate cases in the analysis. One patient experienced a recurrence two months after the initial tumor resection and ND. During the resection of the recurrent tumor, a cLNM was detected. Consequently, the tumor classification after the second procedure was considered the definitive tumor staging.

A structured literature research was conducted to identify papers that performed ND on T1 MSCC. The metastases rate and the number needed to treat (NNT) for ND and END were then calculated.

To assess the representativeness of the examined cases in relation to the overall cohort of SCC of the superior level of the oral cavity diagnosed over the past 20 years, Fisher’s exact test was applied. The number of cases with histopathologically confirmed cLNM in ND and cLNM occurrence during follow-up was then correlated with localization of primary tumor. Fisher’s exact test and the chi-square test (χ²) were used to determine the statistical significance of observed correlations.

Statistical analyses were conducted using Microsoft Excel 2016 (Microsoft, Redmond, WA, USA) and IBM SPSS Statistics^®^ Version 28 (IBM, Armonk, NY, USA). A *p*-value of ≤ 0.05 was considered statistically significant.

The study was approved by the hospital’s ethics committee (No. 10871_BO_K_2023).

## Results

### Research collective

**Fig. 1:**
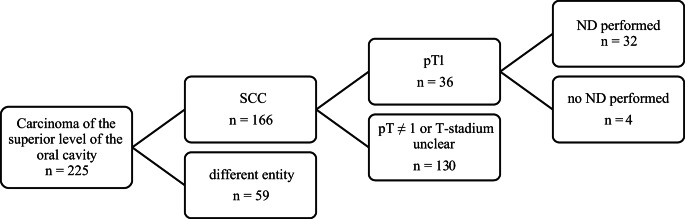
Illustrates the selection of patients included in the study. A total of 225 cases of carcinoma of the superior level of the oral cavity were recorded at MHH between 2003 and 2023, of which 36 patients met the inclusion criteria, 32 of them received ND

Between 2003 and 2023, a total of 225 patients were diagnosed with carcinoma of the superior level of the oral cavity at MHH. The most common histopathologic entity was SCC (*n* = 166, 73.78%), followed by adenoid cystic carcinoma (*n* = 17, 7.56%), mucoepidermoid carcinoma (*n* = 12, 5.33%), and adenocarcinoma (*n* = 11, 4.89%). Among SCC, 36 (21.69%) were classified as pT1. The results of staging examinations, particularly with regard to potential cLNM, were available in 20 cases. In 17 cases (85%), no evidence of cLNM (cN0) was found, whereas 3 cases (15%) showed clinical signs of cLNM. ND was performed in 32 of these cases (86.49%) (see Fig. 1), with 13 conducted as END following prior cN0 staging, and 3 as therapeutic ND (TND) due to evidence of cLNM on staging.

Among the 36 patients included in the study, 19 were women (52.78%) and 17 were men (47.22%), resulting in a male-to-female ratio of 1:1.12. The majority of SCC cases were located on the hard palate, alveolar process and maxillary gingiva (≙ MSCC) (*n* = 25, 69.44%), while fewer cases were found on the soft palate (*n* = 11, 30.56%) (see Table 1).

**Table 1:** Presents a comparison between the investigated cohort and the total group of carcinomas of the superior level of the oral cavity, showing no significant differences between the two

	Study collective		Total				
Feature	*n*	%		*n*	%	*p* [Fisher]	
Gender						*1.00*	
Male	17	47.22		106	47.11		
Female	19	52.78		119	52.89		
Localization						*0.85*	
Hard palate, alveolar process and maxillary gingiva	25	69.44		150	66.8		
Soft palate	11	30.56		75	33.2		
Total	**36**			**225**			

### Neck dissection and cervical lymph node metastases

Of the pT1 SCC cases analyzed, ND was conducted in 32 patients (88.88%), with bilateral ND performed in the majority of cases (*n* = 27, 84.38% of all ND). The most frequently performed ND involved four levels (*n* = 23, 71.88% of all ND) (see Table 2).

**Table 2:** Presents data on the extent of ND, differentiating between unilateral and bilateral procedures, as well as the dissected lymph node levels. Additionally, it outlines the frequency of cLNM, including the localization of the primary tumor and the corresponding pN staging

Feature	*n*	%	*p* [Fisher]
Implementation ND			
Unilateral ND	5	15.63	
Bilateral ND	27	84.38	
Extent of ND (in case of bilateral ND the higher extent)			
LN picking	1	3.13	
3 Level	2	6.25	
4 Level	23	71.88	
5 Level	1	3.13	
6 Level	5	15.63	
**Total**	**32**		
cLNM at the onset of the disease			
no cLNM	31	86.11	
cLNM	5	13.89	
Total of which:	**36**		
pN1	4	80.00	
pN2c	1	20.00	
Localization of the primary tumor in cases of occult metastases			0.023
hard palate and alveolar process	1	20.00	
Soft palate	4	80.00	

In total, cLNM were identified in five cases (13.89%), in one case following unilateral ND and in four cases after bilateral ND (*p* [Fisher] = 0.599). The metastases were found after dissection of three levels (*n* = 1, pN1), four levels (*n* = 3, pN1), and six levels (*n* = 1, pN2c) (*p* [χ²] = 0.671). In one case, cLNM occurred bilaterally (2.78%), while in four cases, they were ipsilateral (11.11%). Four cases were classified as pN1, while one case was classified as pN2c. Among the 25 patients with pT1 MSCC, only one (4.00%) exhibited cLNM. In contrast, SCC of the soft palate was associated with a significantly higher rate of cLNM, detected in four out of eleven cases (36.36%) (*p* [Fisher] = 0.023) (see Table 2). Of the 17 patients staged as cN0, 13 underwent END without any histopathological evidence of cLNM. The remaining 4 patients did not undergo END and showed no signs of cLNM during the follow-up period. Among the patients who presented with signs of cLNM on staging (*n* = 3), all underwent ND at six levels. One patient was found to have pN2c, while two were confirmed as pN0.

### Follow-up

During follow-up, 23 patients continued regular monitoring at MHH until January 1 st, 2023, or until recurrence occurred. The average follow-up duration was 1,421.44 days (minimum: 206 days, maximum: 4,617 days, SD: 1,016.30). Follow-up data were missing for 13 out of 36 patients (36.11%) due to irregular attendance at scheduled appointments or preference for local follow-up care.

Among the patients monitored, one developed regional recurrence on the ipsilateral side 1.8 years after the initial diagnosis (initial pTNM: pT1pN0; primary tumor localization: maxillary gingiva). No occult cLNM had been detected initially, and bilateral ND at four levels had been performed. Additionally, two other patients experienced locoregional recurrence during follow-up: one with an initial pN0 stage and the other with an initial pN1 stage (see Table 3).

**Table 3:** Shows the cases that developed recurrence and summarizes the type and timing of recurrence in relation to the initial pN classification and primary localization. It also details whether ND was performed initially. Three out of 23 patients (13,04%) showed recurrence in the follow-up

Type of recurrence	Initial pN classification	localization of primary	Time until recurrence (days)	initial ND (level/uni- or bilateral)
regional	pN0	m	660	4/bilateral
locoregional	pN0	m	555	6/bilateral
locoregional	pN1	s	2093	4/bilateral

m = maxilla, s = soft palate

## Discussion

The current guideline states that in cases of MSCC with an invasion depth of up to 3 mm, END may be omitted. However, accurately determining invasion depth preoperatively remains challenging. Therefore, statistically reliable data is essential for making well-informed preoperative decisions regarding the necessity of ND.

The data analysis presented above indicates that when SCC is confined to the hard palate, alveolar process and maxillary gingiva, cLNM are extremely rare. This study found a significant difference in the metastatic behavior of pT1 MSCC compared to SCC of the soft palate. These findings clearly demonstrate the necessity of a strict differentiation between MSCC and SCC of the soft palate in the clinical management of SCC involving the superior oral cavity. Previous studies have demonstrated that maxillary carcinoma may also drain into the parapharyngeal sentinel lymph nodes (Boeve et al., 2017). It may be hypothesized that the closer a tumor is situated to the oropharynx, the greater the likelihood of drainage through parapharyngeal lymph nodes, potentially accounting for higher rates of cLNM observed in soft palate SCC.

According to existing literature, END is recommended when the probability of cLNM exceeds 20% [27, 28]. Based on the findings of this study, END may not be necessary for pT1 MSCC but may show advantages for SCC of the soft palate. This recommendation has been deliberately worded with semantic caution, given that only a very small number (n = 11) of pT1 SCC of the soft palate have occurred at MHH over the past 20 years and therefore were available for analysis. However, Moratin et al. argue that END may still offer potential benefits in T1 MSCC; in their study of 24 T1 MSCC cases, they identified occult cLNM in four patients and therefore proclaim a potential advantage of END [16].

It is important to note that this study only included SCC cases that had undergone histopathological analysis, meaning tumor size and, more specifically, depth of invasion were already known. In contrast, preoperative assessment of invasion depth is highly challenging. Staging examinations can only detect bone erosion, which would result in a significantly higher T classification. This complicates decision-making regarding END, particularly in borderline cases. A two-stage approach could be considered in such cases, as it would allow for tumor-free resection margins to be confirmed histopathologically before proceeding with ND and reconstructive surgery. The planning of reconstruction could thus take into account the potential need for adjuvant therapy.

When considering morbidity, it is also crucial to recognize that, surgical morbidity also results from preparing the recipient vessels for microvascular reconstruction. However, in patients who do not require microvascular reconstruction, END may represent a significant additional morbidity burden. Spalthoff et al. could demonstrate that the more extended the ND, the greater the impairment, especially in terms of quality of life [23]. A similar perspective is offered by Beltramini et al., who recommend END only for patients already requiring cervical access for other surgical reasons [29], which as well represents the standard operating procedure in our department. Sentinel lymph node biopsy (SLNB) is a promising addition to the management of the clinically node negative neck in low-stage OSCC reducing morbidity in comparison to ND [30]. It could offer a middle way between watch-and-wait and END and has a negative predictive value of 97%, a false negative rate of 7.8%, and sensitivity of 92% [31]. However, for MSCC, there is very limited evidence regarding the suitability of SLNB, with some authors carefully recommending considering SLNB [32]. The different extent of ND in the present study is most likely attributable to the long observation period (20 years). During this time, different surgeons applied varying concepts regarding cervical lymph node management. This reflects the issue described by Pabst et al., namely that the implementation of ND is inconsistent even among German tumor centers [26].

Data collection and literature review confirm that pT1 SCC of the maxilla and soft palate is rare, with only 36 cases identified at the MHH between 2003 and 2023. Despite this, the present study includes a comparatively large number of pT1 MSCC cases (*n* = 25). In the existing literature, only a few studies report similar case numbers. To our knowledge, a total of 357 cases of cT1/pT1 MSCC have been documented in the literature until now, with 42 (11.76%) showing cLNM - either detected during END/TND or observed during follow-up. This corresponds to a number needed to treat (NNT) of 16 for ND in T1 MSCC (see Table 4) and NNT of 18 for END, further emphasizing the need for larger research cohorts to enable well-founded, evidence-based decision-making regarding ND.

**Table 4:** Provides an overview of MSCC cases from current literature, focusing on the performance of ND and the occurrence of cLNM. The NNT was calculated based on the data presented. The sources for Table 4 are provided in Table 5

	No performance of ND (*n*)	Performance of ND (*n*)	Total (*n*)
no cLNM	93	149	242
cLNM	5	19	24
Total	**98**	**168**	**266**

From the data given in Table 5, a metastases rate of 11.76% is calculated. Given the premise that END can be avoided if the probability of cLNM is below 20%, END for T1 MSCC should be regarded critically. In this case, END appears to cause more harm than benefit. These findings align with those of Feng et al., who found no overall survival advantage associated with END in MSCC in terms of disease-specific survival [15].

**Table 5:** Presents cases of T1 MSCC and the occurrence frequency of cLNM (detected during ND or follow-up period) reported in the current literature. It also documents the performance of END and the frequency of occult cLNM. * highlights the studies that could not be included for calculation in table 4, mostly because it was not clear whether cLNM occurred after ND or during follow-up period

Author	T1 (*n*)	cLNM (*n*)	Of that: ND (*n*)/cLNM (*n*)	Of that: END (*n*)/occult cLNM (*n*)	
[29]	7	0	0/0	0/0	
[33]	34	2	34/2		
[34]	7	1	0/0	0/0	
[35]	1	0	0/0	0/0	
[21]	25	7	12/3		*
[15]	27	4	5/2	5/2	
[19]	8	2	8/2	8/2	
[36]	18	0	12/0	12/0	
[32]	19	0	0/0	0/0	
[37]	6	4	6/4	4/2	
[16]	24	4			*
[38]	38	7			*
[39]	2	0	0/0	0/0	
[40]	75	7	75/7	75/7	
[41]	6	1	2/1	1/0	
[42]	10	0	2/0	2/0	
[43]	14	1	0/0	0/0	
[44]	4	0			*
[45]	7	1	2/0	2/0	
Present study	25	1	22/1	13/0	
Total	**357**	**42**	**168/19**	**122/13**	

A key challenge in acquiring sufficient data is the rarity of T1 MSCC, making it difficult for individual centers to collect large datasets. A multi-center study or meta-analysis could help clarify this issue and provide more reliable data regarding the necessity of END in T1 MSCC.

As outlined in the current German guidelines, *“the evidence is not sufficient to make a general recommendation against ND [.] or in favor of a ‘wait and see’ approach”* [25]. According to the findings of this study this highlights the need for data and the authors encourage all surgeons involved in maxillary cancer treatment in gathering data to establish an empirically based consensus on END in T1 MSCC.

## Data Availability

The data that support the findings of this study are not openly available due to reasons of sensitivity and are available from the corresponding author upon reasonable request. Data are located in controlled access data storage at MHH.
